# The impact of propolis on clinical manifestations and specific IgE levels against salsola in perennial-allergic rhinitis patients

**DOI:** 10.5339/qmj.2023.sqac.21

**Published:** 2023-11-26

**Authors:** Farahzad Jabbari-Azad, Maryam Khoshkhui, Negar Khalighi, Mojgan Mohammadi, Maryam Emadzadeh, Mehraneh Movahedi Aliabadi

**Affiliations:** ^1^Allergy Research Center, Mashhad University of Medical Sciences, Mashhad, Iran Email: Jabbarif@mums.ac.ir; ^2^Immunology Research Center, Mashhad University of Medical Sciences, Mashhad, Iran; ^3^Clinical Research Development Unit, Ghaem Hospital, Mashhad University of Medical Sciences, Mashhad, Iran

## Abstract

**Objectives:** Propolis has an anti-inflammatory effect induced by inhibiting cyclooxygenase, subsequent inhibition of prostaglandin and nitric oxide synthesis, reduction of inflammatory cytokines, and eventually immunosuppressive activity [1-3]. This study aims to evaluate the impact of propolis on clinical features and specific IgE levels against salsola in perennial allergic rhinitis patients.

**Methods:** Thirty patients diagnosed with perennial allergic rhinitis with salsola-positive skin prick test were enrolled in this randomized controlled clinical trial and divided into two groups. The intervention group received the propolis (200 mg per day), and the control group received a placebo for four months, besides intranasal corticosteroids. At baseline and the end of the intervention, the level of Salsola-specific IgE was measured by the RAST method. To assess the propolis effect on the quality of life and disease severity, miniRQLQ and SNOT22 questionnaires were completed by patients before and after the intervention.

**Results:** According to Table 1, Serum IgE level showed decreasing changes (-0.057) despite increasing changes in the control group (1.039). However, these differences were not statistically significant (P = 0.967). Based on the miniRQLQ questionnaire, quality of life improved in both groups without any significant difference (P = 0.930). According to the SNOT-22 questionnaire, both groups’ nasal and sinus problems decreased significantly. However, the intervention type did not affect this decrease and was observed over time in both groups (P> 0.05).

**Conclusion:** Propolis supplementation did not significantly affect various laboratory parameters, clinical symptoms, and quality of life of patients with allergic rhinitis.

## Figures and Tables

**Table 1.** Serum Ige Level in Control and Intervention Groups

	**Serum IgE Level (KU/L)**			**IgE Level Difference (KU/L)**
**Groups**	**Baseline**	**Post-treatment**	**P-value**	
** Control**	1.59±1.11	4.47±1.94	0.959	3.15±1.04
** Intervention**	0.92±0.81	0.87±0.62	0.767	-0.86±0.057
** P-value**	0.706	0.504		0.967
